# Disinhibition-assisted long-term potentiation in the prefrontal-amygdala pathway via suppression of somatostatin-expressing interneurons

**DOI:** 10.1117/1.NPh.7.1.015007

**Published:** 2020-02-14

**Authors:** Wataru Ito, Brendon Fusco, Alexei Morozov

**Affiliations:** a Fralin Biomedical Research Institute at VTC, Roanoke, Virginia, United States; b Virginia Tech, School of Biomedical Engineering and Sciences, Blacksburg, Virginia, United States; c Virginia Tech Carilion School of Medicine, Department of Psychiatry and Behavioral Medicine, Roanoke, Virginia, United States

**Keywords:** long-term potentiation, disinhibition, amygdala, somatostatin interneurons

## Abstract

**Significance:** Natural brain adaptations often involve changes in synaptic strength. The artificial manipulations can help investigate the role of synaptic strength in a specific brain circuit not only in various physiological phenomena like correlated neuronal firing and oscillations but also in behaviors. High- and low-frequency stimulation at presynaptic sites has been used widely to induce long-term potentiation (LTP) and depression. This approach is effective in many brain areas but not in the basolateral amygdala (BLA) because the robust local GABAergic tone inside BLA restricts synaptic plasticity.

**Aim:** We aimed at identifying the subclass of GABAergic neurons that gate LTP in the BLA afferents from the dorsomedial prefrontal cortex (dmPFC).

**Approach:** Chemogenetic or optogenetic suppression of specific GABAergic neurons in BLA was combined with high-frequency stimulation of the BLA afferents as a method for LTP induction.

**Results:** Chemogenetic suppression of somatostatin-positive interneurons (Sst-INs) enabled the *ex vivo* LTP by high-frequency stimulation of the afferent but the suppression of parvalbumin-positive interneurons (PV-INs) did not. Moreover, optogenetic suppression of Sst-INs with Arch also enabled LTP of the dmPFC-BLA synapses, both *ex vivo* and *in vivo*.

**Conclusions:** These findings reveal that Sst-INs but not PV-INs gate LTP in the dmPFC-BLA pathway and provide a method for artificial synaptic facilitation in BLA.

## 1 Introduction

Optogenetics and chemogenetics have become the key methods for testing the causal role of specific neuronal populations and synapses in brain activities and animal behaviors. The techniques employ depolarizing or hyperpolarizing neuronal compartments, like the soma, dendrites, and synaptic terminals, to trigger or suppress action potentials (APs) and release of neurotransmitters.^1^^,^^2^ Meanwhile, natural neuronal adaptations driven by experience and learning, or observed during development or in disease, involve brain alterations, not only in neuronal activity but also in synaptic efficacy. Modeling and quantitative analyses of such naturally occurring brain adaptations require techniques for selective manipulation of synaptic strength, both *ex vivo* and *in vivo*. In many cases, synapses can be potentiated or depressed by applying high- or low-frequency presynaptic stimulation, respectively.^3^ With optogenetic stimulation, this simple approach proved successful in several circuits *ex vivo* and *in vivo*. The 20 Hz and theta-burst stimulation produced long-term potentiation (LTP) in the recurrent synapses of the hippocampal area CA3^4^ and corticostriatal synapses,^5^ respectively. In contrast, the low-frequency stimulation produced long-term depression (LTD) in the inputs to the nucleus accumbens and basolateral amygdala (BLA) from the infralimbic cortex.^6^^,^^7^ However, not all synapses follow the frequency rule. For example, the high-frequency stimulation generated LTD in inputs from the BLA to the dorsomedial prefrontal cortex (dmPFC)^8^ and failed to produce LTP in the prefrontal-amygdala synapses.^9^

Evidence accumulates that the strength of remote synaptic inputs to the BLA is a critical determinant of fear behaviors and readily changes by emotional experiences. Specifically, auditory fear conditioning, in which the animal experiences a neutral conditioned stimulus followed by electrical footshocks as the unconditioned stimulus (US), facilitates inputs in the lateral subdivision of BLA from the sensory cortex and sensory thalamus.^10^^,^^11^ Conversely, optogenetically induced LTD and LTP in the same pathway deactivate and reactivate fear memories, respectively.^12^

Besides the sensory inputs, BLA receives abundant inputs from the dmPFC,^13^ and the properties of the dmPFC-BLA reciprocal circuit have been correlated with distinct emotional states. Specifically, the fear extinction training has been found to depress the prefrontal-amygdala synapses and to strengthen the reciprocal amygdala-prefrontal synapses.^14^^,^^15^ The pattern of neuronal synchronization between dmPFC and BLA has been found to determine whether the animal expresses defensive behaviors or feels safe,^16^^,^^17^ suggesting that the strength of synaptic connections in the dmPFC-BLA reciprocal circuit regulates the emotional state. Testing this prediction would require artificial facilitation or depression of the prefrontal-amygdala synapses. However, there are no reports of artificially induced LTP in the prefrontal-amygdala synapses, likely because the local GABAergic neurons provide potent feedforward inhibition and gate plasticity in the remote glutamatergic inputs.^18^^,^^19^ Meanwhile, the robustness of the “natural” behavior-driven plasticity is explained by the US actions to disinhibit the BLA by attenuating GABAergic transmission via multiple mechanisms, including the secretion of neuromodulators.^19^^–^^21^

Here, to achieve reliable artificial LTP inductions in the BLA input from dmPFC, we tested the effects of chemogenetic/optogenetic suppression of GABAergic transmission during the LTP induction by high-frequency stimulation. The two major classes of GABAergic neurons—the parvalbumin-positive interneurons (PV-INs) and somatostatin-positive interneurons (Sst-INs)^22^—were suppressed individually, which revealed that the Sst-INs gate the artificially induced LTP.

## 2 Materials and Methods

### 2.1 Animals

All mice were either wild type or transgenic males on the 129SvEv/C57BL/6N F1 hybrid background. The experiments were limited to the male mice, to avoid potential sex-dependent LTP variability, which has been reported both in the amygdala^23^^,^^24^ and hippocampus.^25^^,^^26^ To obtain the mice-expressing hM4Di^27^ in Sst-INs or PV-INs, homozygous R26-LSL-Gi-DREADD males (JAX Stock No: 026219) on C57BL/6N background were crossed with homozygous interneuron-specific Cre driver females on 129SvEv background—either the Sst-IN-specific Cre driver (Sst-Cre), $Sst^{tm2.1(cre)Zjh}$^28^ (JAX: 013044) or the PV-IN-specific Cre driver (PV-Cre), $Pvalb^{tm1(cre)Arbr}$^29^ (JAX: 008069). The expression specificity of floxed reporters in the BLA of these driver lines has been validated using immunostaining and whole-cell recordings by the Luthi lab.^22^ To obtain heterozygous Sst-Cre mice, wild type C57BL/6N males were crossed with the 129SvEv homozygous $Sst^{tm2.1(cre)Zjh}$ females. All breeding were the trios of one male and two females on the C57BL/6N and 129SvEv backgrounds, respectively. Male pups were weaned at p21–p25 and housed three to five littermates per cage. All experiments were approved by Virginia Tech IACUC and followed the NIH Guide for the Care and Use of Laboratory Animals.

### 2.2 Surgery—Viral Injection and Optrode Implantation

Adenoassociated viruses (AAVs) for expressing Chronos or Cre-activated Arch were generated from pAAV-Syn-Chronos-GFP^30^ (Addgene #59170) or pAAV-FLEX-Arch-GFP (Addgene #22222), respectively, gifts from Edward Boyden. The viruses (pseudotype 5 for Chronos and pseudotype 1 for Arch) were prepared by the University of North Carolina Vector Core (Chapel Hill, North Carolina). At p28, the heterozygous Sst-Cre male mice were anesthetized by intramuscular injection of ketamine/xylazine/acepromazine, 100/5.4/1  mg/kg, as routinely done for electrode implantations in the Buzsaki lab,^31^ in the volume not exceeding 0.05 ml, placed in a stereotaxic apparatus (David Kopf, Tujunga, California) and underwent minimum craniotomy (∼0.5  mm diameter). For the dmPFC virus injection, the dura mater was preserved. A heater-pulled short-taper glass pipette (shaft: 0.6/0.4  mm external/internal diameter, beveled tip: 50  μm, diameter, Drummond, Broomall, Pennsylvania) filled with the virus solution ($10^{12}\;\text{viral particles}/\mathrm{ml}$) was slowly lowered to the target (1.3 mm anterior, 0.4 mm lateral from bregma, and 1.3 mm ventral from brain surface). The solution (0.5  μl) was injected bilaterally at the rate of 0.2  μl/min using a syringe pump connected to the pipette through plastic tubing filled with water as described.^32^ For the BLA virus injections, the dura mater was removed to allow straight penetration by a less rigid long-taper pipette. The virus solution (0.4  μl, $10^{12}\;\text{particles}/\mathrm{ml}$) was injected bilaterally at the rate of 0.1  μl/min at the coordinates (1.2 mm posterior, 3.2 mm lateral from bregma, and 4.2 mm ventral from brain surface). Custom-made optrodes were fabricated with a miniature LED [C460EZ500, CREE, peak wavelength 463.3 nm, bandwidth 20 nm full-width half maximum (FWHM)], coupled to an optical fiber (0.66 NA, 0.2-mm core diameter, Prizmatix) with UV-cured index-matched glue (Norland Optical Adhesive 85, Norland) and two 33-μm tungsten wires (polyimide-insulated, California Fine Wire) extended 0.5 mm beyond the optical fiber. The optrodes for *in vivo* recording were implanted in BLA at p60, as described.^33^ For postoperation analgesia, ketoprofen (5  mg/kg) was administered subcutaneously.

### 2.3 *Ex Vivo* Recordings

#### 2.3.1 General

Mice were anesthetized with intraperitoneal injection of avertin, 0.4  mg/kg, and intracardially perfused with ice-cold partial sucrose artificial cerebrospinal fluid (ACSF) solution containing (in mM) 80 NaCl, 3.5 KCl, 4.5 ${\mathrm{MgSO}}_{4}$, 0.5 ${\mathrm{CaCl}}_{2}$, 1.25 $H_{2}{\mathrm{PO}}_{4}$, 25 ${\mathrm{NaHCO}}_{3}$, 10 glucose, and 90 sucrose equilibrated with 95% $O_{2}/5\%$ ${\mathrm{CO}}_{2}$.^34^ Amygdala slices, 300  μm thick, were prepared at the angle of 35 deg from horizontal [Fig. 4(c)] and stored, as described earlier.^33^ Recording chamber was superfused at 2  ml/min with ACSF equilibrated with 95% $O_{2}/5\%$ ${\mathrm{CO}}_{2}$ and containing (in mM) 119 NaCl, 2.5 KCl, 1 ${\mathrm{MgSO}}_{4}$, 2.5 ${\mathrm{CaCl}}_{2}$, 1.25 $H_{2}{\mathrm{PO}}_{4}$, 26 ${\mathrm{NaHCO}}_{3}$, and 10 glucose (pH 7.4), and maintained at 30±1°C. Whole-cell recordings were obtained with EPC-10 amplifier and Pulse v8.76 software (HEKA Elektronik, Lambrecht/Pfalz, Germany). Putative glutamatergic neurons in BLA were identified by their pyramidal morphology^35^ under Dodt gradient contrast optics (custom made) at 850-nm LED illumination (Thorlabs, Newton, New Jersey). GABAergic neurons expressing hM4Di-Citrine or Arch-GFP were identified by fluorescence. The recording pipettes (3 to 5  MΩ) were filled with (in mM) 120 K-gluconate, 5 NaCl, $1\;{\mathrm{MgCl}}_{2}$, 10 HEPES, 0.2 EGTA, 2 ATP-Mg, and 0.1 GTP-Na for current-clamp recordings or with 120 Cs-methanesulfonate, 5 NaCl, $1\;{\mathrm{MgCl}}_{2}$, 10 HEPES, 0.2 EGTA, 2 ATP-Mg, 0.1 GTP-Na, and 10 mM QX314 for voltage-clamp recordings. Both internal solutions were set at pH 7.3 and osmolarity 285 Osm. Membrane potentials were corrected by the junction potential of 12 mV. Series resistance (Rs) was 10 to 20  MΩ and monitored throughout experiments to exclude the recording data if the Rs changed more than 20%. LFP recordings were obtained using Multiclamp 700B amplifier and Digidata 1440A (Molecular Devices, Sunnyvale, California). The recording pipettes (1 to 2  MΩ) were filled with ASCF. Blue and yellow light beams from a blue LED [M470L2, Thorlabs, peak wavelength 470 nm, bandwidth 25 nm (FWHM)] and a yellow LED (Luxeon Rebel PC Amber, Luxeon Star LEDs, peak wavelength 591 nm, bandwidth 55 nm) were combined with a dichroic mirror (FF562-Di03, Semrock) and injected to the epifluorescence excitation light path of the scope (BX51WI, Olympus, Center Valley, Pennsylvania). Light pulses of each color were generated using custom LED drivers based on MOSFET and were delivered through a 40× objective lens (Olympus) at the irradiance of 0.5 to $5\;{\mathrm{mW}/\mathrm{mm}}^{2}$, calibrated by a photodiode power sensor (Thorlabs) at the tip of the lens.

#### 2.3.2 LTP

In both the whole-cell and LFP recording, the strength of test pulses (1 ms duration) was adjusted to elicit responses at 30% to 40% of the maximum. In the whole-cell recordings, test pulses were given every 30 s. LTP was induced by six 2-s trains of 50 Hz and 1 ms pulses. The trains were given at the 10-s interval [Fig. 2(a)]. In the LFP recordings, test pulses were given every 20 s. LTP was induced using the “spaced protocol.” It included pairs of 1-s trains of 50 Hz and 1 ms pulses, separated by 10 s. The pairs were repeated five times at the 3-min interval [Fig. 3(a)]. This protocol is the same as in a published study on LTP in BLA,^36^ except the stimulation frequency was decreased from 100 to 50 Hz to allow reliable activation of Chronos.^30^ In some experiments, continuous yellow light was given during the trains of the blue light pulses. The yellow light strength was set below the levels that trigger the release of glutamate from the axonal terminals expressing Chronos (data not shown).

### 2.4 *In Vivo* Recordings

The subject animals, bilaterally injected with the AAV-Chronos in the dmPFC, AAV-Arch in BLA, and bilaterally implanted with the optrodes in the BLA, were housed with the littermates until the experiment. Using the RHA2000-Series Amplifier USB Evaluation Board (RHA2000-EVAL, Intan Technologies), the local field potentials (LFPs) were recorded from BLA of the subject mouse in the home cage without the lid, from where the cagemates were removed temporarily for the duration of the recording. Mice were habituated to the recording environment by connecting to the recording system for 2 to 3 h per day during 2 to 3 consecutive days. Field excitatory postsynaptic potentials (fEPSPs) were elicited in BLA by blue light stimulation of dmPFC terminals expressing Chronos. The strength of the test pulses (1 ms, 2 to 3 mW at the tip of optrode) was adjusted to obtain the fEPSP slope at 30% to 40% of the maximum. The LED driver (PlexBright LD-1, Plexon) was analog-modulated by DAQ (Analog Shield, Digilent). The LED driving current was routed to the optrodes in either hemisphere by electrical relays (Arduino 4 relays shield, Arduino). Arduino with a custom Arduino sketch controlled both the DAQ and the relays to give the light stimulation on each side every 30 s, alternating the sides every 15 s. Once the baseline of evoked fEPSPs stabilized, LTP was induced by the same blue light stimulation protocol as in the LFP and LTP experiments *ex vivo* [Fig. 3(a)], except that the protocol was repeated three times for every 1 h. The positions of the optrodes were confirmed by histological analysis.

### 2.5 Immunofluorescence

Mice were anesthetized with an intraperitoneal injection of avertin, 0.4  mg/kg, and intracardially perfused with paraformaldehyde (PFA, 4%). Brains were postfixed in PFA overnight and sliced into 75  μm sections. The slices were stained using a rat monoclonal Sst antibody MAB354 (Millipore) dilution 1:600, followed by a Cy3 conjugated donkey antirat antibody (1:400) and imaged using Zeiss LSM710 confocal microscope.

### 2.6 Data Analysis

Data were processed using custom scripts written in MATLAB (MathWorks) and Clampfit software (Molecular Devices). Statistical analyses were performed using GraphPad Prism 5 (GraphPad Software, La Jolla, California). Normality was tested using the Shapiro–Wilk test. Datasets with normal distribution were compared using the one-sample t-test. The datasets with non-normal distribution were analyzed using the Mann–Whitney test and the Wilcoxon’s signed rank test. The difference was deemed significant with p<0.05.

## 3 Results

### 3.1 DREADD-hM4D(Gi) Suppresses GABA Release from BLA Interneurons

**Fig. 1:**
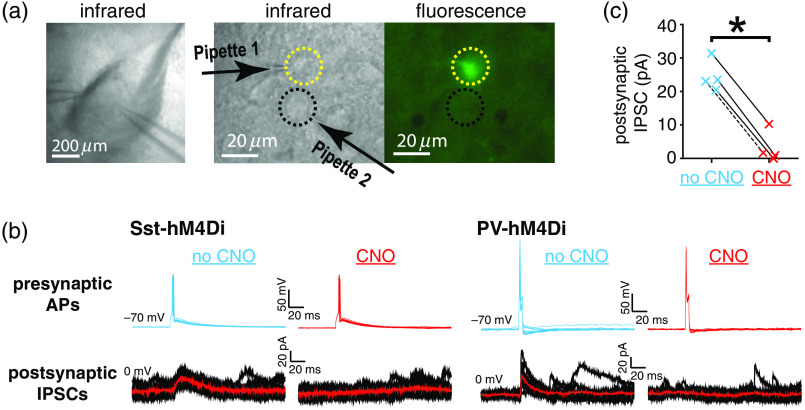
DREADD suppresses GABA release from BLA INs during APs. (a) Example of the paired whole-cell recording from a BLA slice. (a) Left: Infrared (IR) image at low magnification. Right: high magnification IR and fluorescent images. Dotted yellow and black circles indicate an Sst-IN expressing hM4D(Gi)-citrine identified by fluorescence and a putative PN, respectively. (b) Examples of double patch recordings of APs evoked by current injection in the INs (400 pA, every 15 s) (upper) and of the corresponding IPSCs in the connected PNs (lower), in the absence of CNO (no CNO, blue) and after 10 min perfusion with 1  μM CNO (CNO, red). Fifteen traces and IPSC averages (red line) are shown for a pair with an Sst-IN [left, Sst-hM4(Di)] and a pair with a PV-IN [right, PV-hM4(Di)]. (c) Summary data for IPSC amplitudes (n=4, including three pairs with Sst-INs, data point connected with continuous lines, and one pair with PV-IN, data points connected with dashed line) in the absence and presence of CNO. *p<0.05, Mann–Whitney test.

The efficiency of DREADD suppression was tested by double-patch recording from connected pairs of an IN-expressing hM4Di-Citrine identified by the fluorescence and a putative principal neuron (PN). The brief depolarizing current was injected in the IN to trigger single AP. It resulted in the inhibitory postsynaptic current (IPSC) in the connected PN, including clozapine-*N*-oxide (CNO) (1  μM) in the bath did not prevent APs but diminished IPSCs (Fig. 1). The DREADD suppression of presynaptic GABA release despite the presence of AP was consistent with published findings.^37^

### 3.2 Chemogenetic Suppression of Sst-INs Enables LTP Induction *Ex Vivo*

**Fig. 2:**
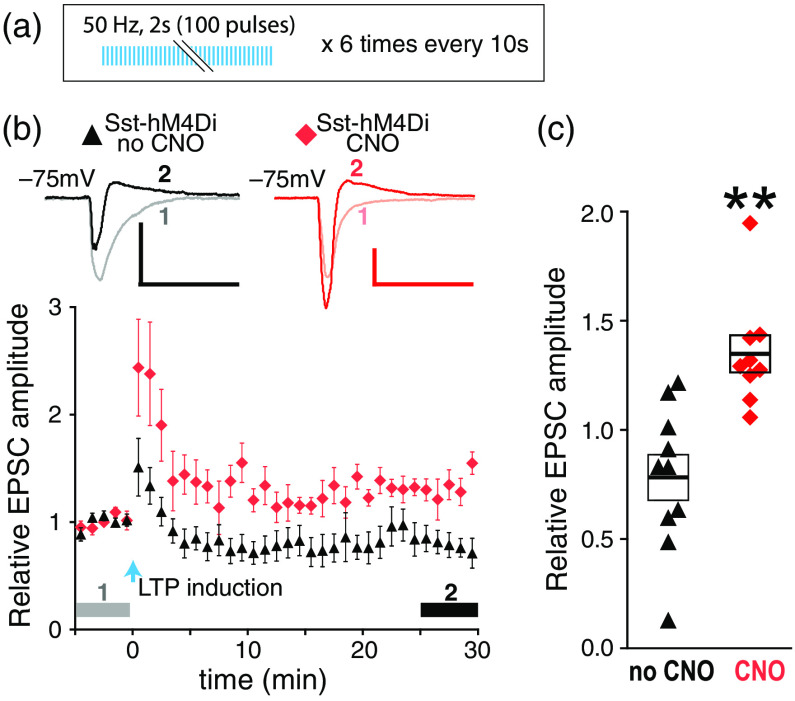
DREADD suppression of Sst-INs enables the facilitation of EPSCs in dmPFC-BLA pathway. (a) LTP induction protocol and (b) relative EPSC amplitudes. Symbols (black triangles: no CNO, red diamonds: CNO) represent the average amplitudes of two consecutive EPSCs recorded during each minute. Upper insets: examples of averaged EPSCs before (1) and after (2) LTP induction as indicated by horizontal gray and black bars, respectively. Scales: 100 pA, 50 ms. (c) Relative EPSC amplitudes averaged during (2) for each neuron. n=10  cells/slices from six mice (no CNO) and nine cells/slices from five mice (CNO). **p<0.01, compared to 1, which is the averaged relative EPSC amplitude during the baseline indicated by gray bars (1), one-sample t-test. Error bars on panel (b) represent standard error of the mean (SEM). Boxes and the thick bars inside on panel (c) represent SEM and means.

For faithful activation of dmPFC axons at high frequency, a fast opsin Chronos^30^ was expressed in dmPFC. The 50-Hz trains of light pulses were used for LTP induction [Fig. 2(a)]. First, we examined the effect of DREADD suppression of Sst-INs on LTP by whole-cell recording from PNs in BLA slices expressing hM4Di in Sst-INs. In the absence of CNO, the 50-Hz stimulation of dmPFC axons caused a brief post-tetanic potentiation of the excitatory postsynaptic currents (EPSCs), followed by a rapid EPSC decline with a tendency toward depression at the 25 to 30 min after the induction (p=0.068, one-sample t-test). In the presence of CNO, the stimulation caused increases in EPSCs lasting for at least 30 min (Fig. 2), suggesting that suppression of Sst-INs enables LTP induction.

**Fig. 3:**
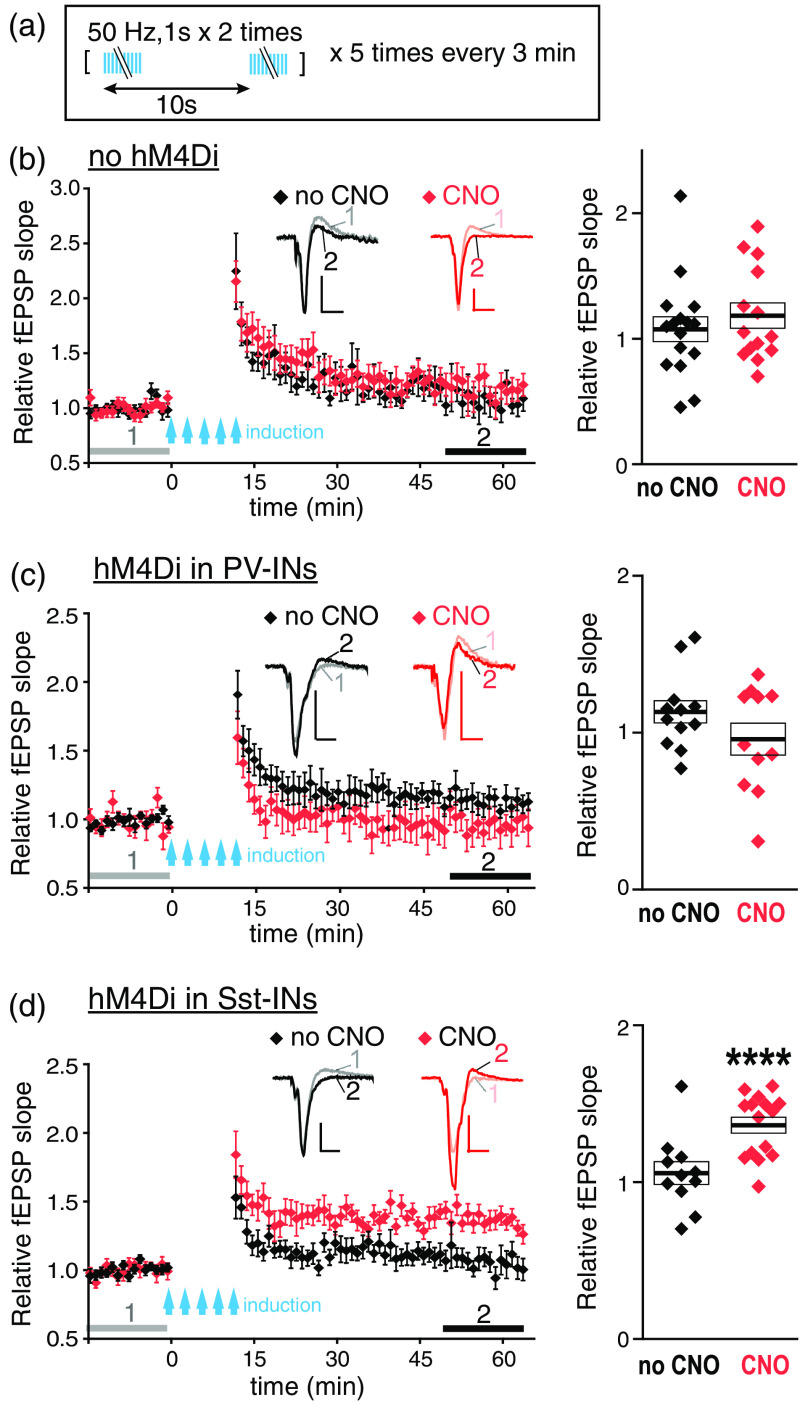
DREADD suppression of Sst-INs enables facilitation of LFPs evoked in the dmPFC-BLA pathway. (a) LTP induction protocol. (b–d) LTP experiments on slices without hM4Di (b), with hM4Di expressed in PV-INs (c), and with hM4Di expressed in Sst-INs (d). Left: relative fEPSP slopes. Symbols on the diagram represent the averages of three consecutive data points obtained every 20 s. Insets represent examples of averaged fEPSPs before (1) and after (2) LTP induction, as indicated by horizontal gray and black bars. Scales: 0.1 mV, 10 ms. Right: relative fEPSP slopes averaged during (2) for each slice. n=16 slices from four mice (no CNO) and 14 slices from five mice (CNO) in (b). n=12 slices from four mice (no CNO) and 11 slices from four mice (CNO) in (c). n=11 slices from three mice (no CNO) and 15 slices from six mice (CNO) in (d). ****p<0.0001, compared to 1, which is the averaged relative fEPSP slope during the baseline indicated by gray bars (1), one-sample t-test. Boxes and the thick bars inside represent SEM and means.

Repeated neuronal stimulation over extended time intervals, or the spaced LTP protocols, induces LTP more effectively than the shorter protocols, which is consistent with greater efficiency of spaced over massed training.^38^ However, our attempts to induce LTP with a spaced protocol during the whole-cell recording have failed (data not shown), presumably because the dialysis of the intracellular content limits the time between obtaining the whole-cell configuration and effective LTP induction. To overcome this limitation, in the following experiments, LTP was tested by recording LFPs and using the spaced LTP protocol [Fig. 3(a)]. We run the CNO control and then tested the effects of suppressing PV-INs, and re-examined the effect of suppressing Sst-INs.

For CNO control, we recorded from slices that did not express hM4Di. There was no significant LTP in the absence or presence of CNO, but there was a tendency toward LTP with CNO [p=0.09, one-sample t-test, Fig. 3(b)]. In slices with hM4Di in PV-INs, there was no significant LTP in the absence or presence of CNO but a tendency toward LTP in the absence of CNO [p=0.09, one-sample t-test, Fig. 3(c)]. In slices expressing hM4Di in Sst-INs, there was a significant LTP in the presence of CNO and no LTP in the absence of CNO [Fig. 3(d)]. These data indicate that (a) CNO in the absence of hM4Di has a minor effect if any on LTP induction, (b) suppression of PV-INs does not aid LTP induction but may rather impede it, and (c) suppression of Sst-INs enables LTP.

### 3.3 Arch in Sst-INs Enables LTP Induction *Ex Vivo* and *In Vivo*

**Fig. 4:**
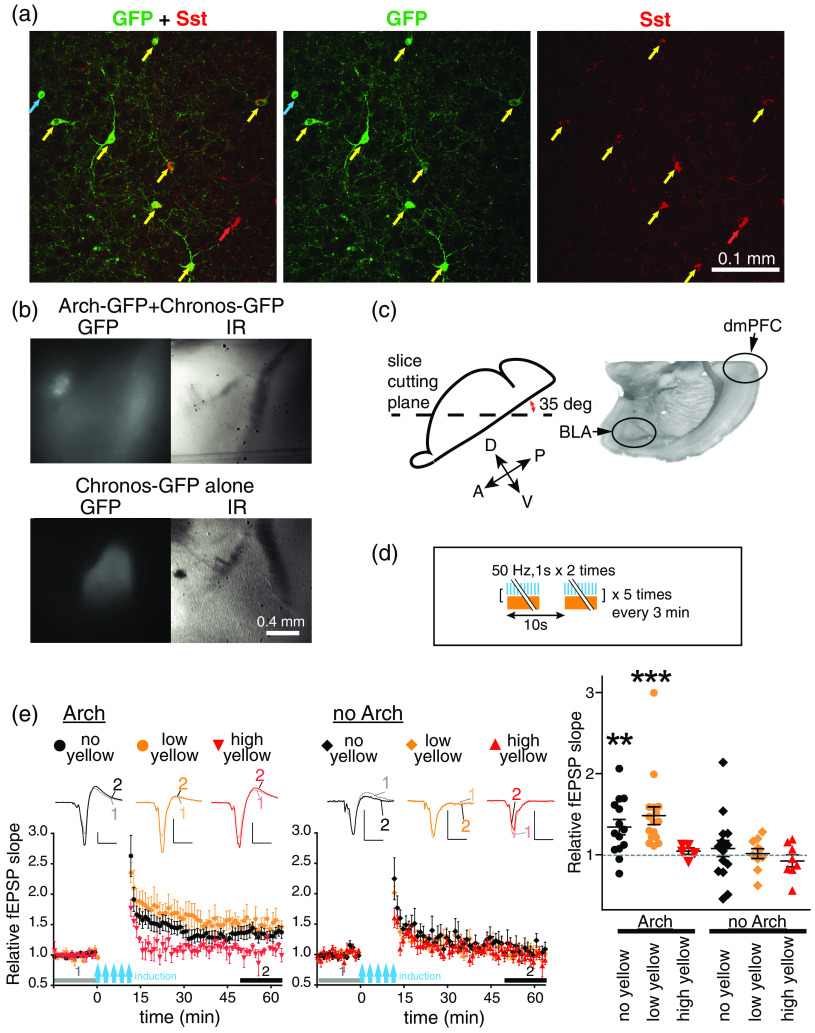
Arch suppression of Sst-INs enables LTP induction. (a) Arch-GFP is expressed mostly in Sst-INs of BLA of an Sst-Cre driver mouse transduced with the floxed Arch AAV in BLA. Confocal image of the somatostatin immunostaining (right panel, Sst, red), Arch-GFP fluorescence (center, GFP, green) and the merged image (left, GFP + Sst). Yellow arrows indicate coexpression of Arch-GFP and Sst, red—Sst only, blue—Arch-GFP only. (b) Examples of slices used for recording. Upper: BLA slice from an Sst-Cre driver mouse transduced with Chronos-AAV in dmPFC and floxed Arch AAV in BLA. Lower: the slice from a mouse transduced only with Chronos-AAV in dmPFC. Fluorescent (GFP) and IR images of the same slices are shown. (c) Left: slice cutting plane. Right: a visible light image of a fixed 150-μm BLA slice cut under the same angle as the 300  μm “live” slices used for recording. (d) LTP induction protocol. (e) LTP experiments on slices with Arch (left) and without Arch (middle) in Sst-INs, with LTP induced using pulses of blue light alone (black circle: no yellow) or combined with the continuous yellow light of two intensities: $0.15\;{\mathrm{mW}/\mathrm{mm}}^{2}$ (orange circles: low yellow) and $0.24\;{\mathrm{mW}/\mathrm{mm}}^{2}$ (red inverted triangles: high yellow). Insets represent examples of averaged fEPSPs before (1) and after (2) LTP induction as indicated by horizontal grey and black bars. Scales: 0.2 mV, 10 ms. Right: Summary data for relative fEPSP slopes averaged during (2) for each slice. n=14 slices from 4 mice (Arch-no yellow), n=17 slices from 10 mice (Arch-low yellow), n=5 slices from 3 mice (Arch-high yellow), n=16 slices from 4 mice (no Arch-no yellow), n=10 slices from 5 mice (no Arch-low yellow), and n=8 slices from 7 mice (no Arch-high yellow). **p<0.01, ***p<0.001, compared to 1, which is the averaged relative fEPSP slope during the baseline indicated by gray bars (1), Wilcoxon’s signed rank test. Boxes and the thick bars inside represent SEM and means.

To examine the effects of optogenetic suppression of Sst-INs, Arch-GFP was expressed in the Sst-neurons of BLA [Figs. 4(a) and 4(b)]. Immunostaining revealed that 82±18% of Arch-GFP expressing neurons were costained with the anti-Sst antibody, and 65±11% of Sst-positive neurons expressed Arch-GFP (n=8 BLA slices from two mice). Presynaptic stimulation was given through Chronos expressed in dmPFC terminals in the same way as in the DREADD suppression experiments. Paired recordings confirmed that Arch attenuated GABAergic transmission between Sst-IN and PN in BLA (Supplemental Fig. S1).

LTP induction in the dmPFC-BLA input was tested by giving trains of blue light pulses alone or combined with the continuous yellow light of different intensities [Fig. 4(d)]. The yellow light by itself did not cause the release of glutamate from the dmPFC axonal terminals in BLA (data not shown). Unexpectedly, the trains of blue light in the absence of yellow light induced a significant LTP [Fig. 4(e), left, black-filled circles]. Combining the trains of blue light and the yellow light of low intensity ($0.15\;{\mathrm{mW}/\mathrm{mm}}^{2}$) produced more significant LTP and with a tendency to be higher than with the blue light alone [Fig. 4(e), left, orange-filled circles]. Increasing the yellow irradiance to $0.24\;{\mathrm{mW}/\mathrm{mm}}^{2}$ prevented LTP induction [Fig. 4(e), left, red-filled inverted triangles]. In slices without Arch, the low-intensity or the high-intensity yellow light did not enable LTP induction by the pulses of blue light [Fig. 4(e), middle]. Together, these data indicate that Arch enables LTP induction by the trains of blue light, and it occurs even in the absence of yellow light, suggesting that the blue light inhibits Sst-INs expressing Arch. Consistently, whole-cell recordings from an Sst-IN with Arch revealed hyperpolarizing currents elicited by blue light (Supplemental Fig. S2). We also found that continuous yellow light at $0.24\;{\mathrm{mW}/\mathrm{mm}}^{2}$ inhibited EPSCs evoked in BLA neurons by stimulating Chronos-expressing dmPFC axons with pulses of blue light (Supplemental Fig. S3). It may result from the desensitization of Chronos by yellow light and would explain the failure of LTP induction in the presence of a stronger yellow light.

**Fig. 5:**
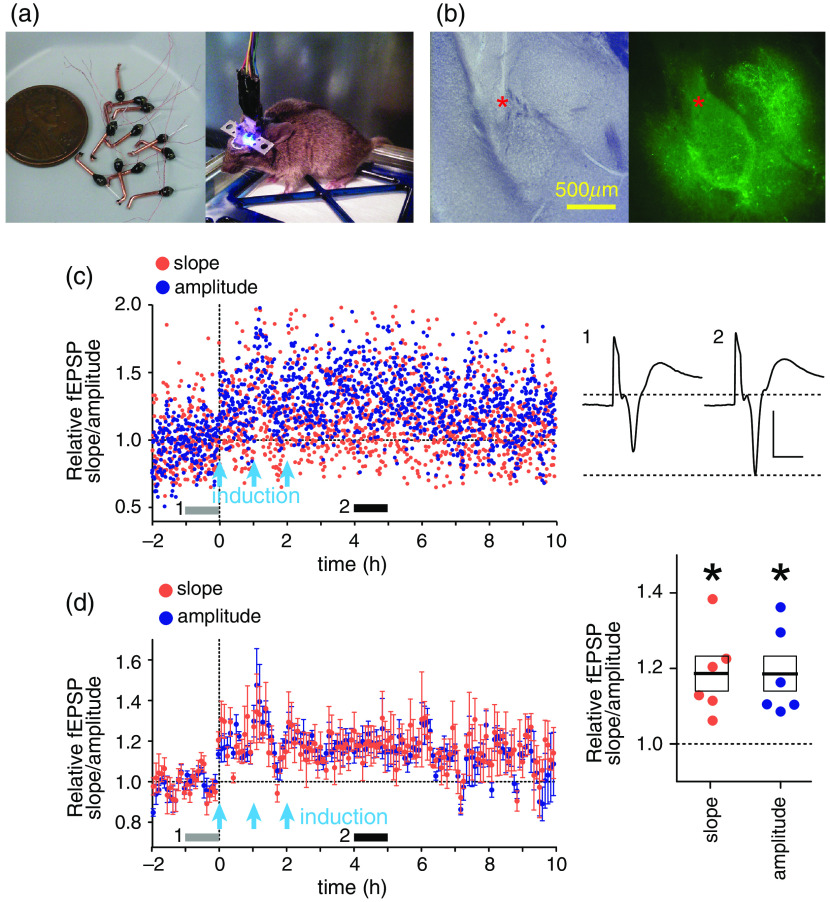
Arch-assisted LTP induction *in vivo*. (a) Left: LED light source-optrode assemblies. Right: a mouse implanted with two optrodes aiming bilaterally at BLA. (b) An example position of electrodes (red asterisks) in a BLA slice imaged under the visible (left) or fluorescent (right) light. The fluorescence arises from Chronos-GFP in dmPFC axons and Arch-GFP in Sst-INs. (c) An example of an LTP experiment showing the slope (red) and amplitude (blue) of light-evoked fEPSP. Light-blue arrows show the trains of 50-Hz light stimulation. The horizontal gray/black bars indicate the ranges for averaging for the sweeps shown on the right (1, gray, 1 h before LTP induction; 2, black, the 3 h after the end of LTP induction). Scales: 0.4 mV, 5 ms. (d) Left: summary LTP experiment. Each data point represents a single independently tested amygdala (n=6 amygdalae from three mice). Right: summary data for the relative fEPSP slope and amplitude facilitation during the 3 h after the end of LTP induction, identified by the horizontal black bar (2). *p<0.05, compared to 1, which is the averaged relative fEPSP slope/amplitude during the baseline indicated by gray bars (1), Wilcoxon’s signed rank test.

To test LTP induction *in vivo*, mice expressing Arch in the BLA Sst-INs and Chronos in dmPFC were implanted with optrodes, whose two electrodes were positioned in BLA and an optical fiber above BLA [Figs. 5(a) and 5(b)]. The LTP induction protocol, identical to the protocol used *ex vivo*, but repeated three times with 1-h interval, produced LTP, which lasted for almost 10 h [Figs. 5(c) and 5(d)].

## 4 Discussion

This study was aimed at developing an efficient protocol for the artificial induction of one form of neuronal plasticity, LTP, in the dmPFC-BLA synapses. This study has three findings: (1) Sst-INs but not PV-INs gate LTP in the dmPFC-BLA pathway induced by high-frequency presynaptic stimuli, (2) removal of the inhibition from Sst-INs, either chemogenetically or optogenetically, both enable the artificial facilitation of this pathway *ex vivo* and *in vivo*, and (3) blue light alone is sufficient for the optogenetically assisted facilitation because the wavelength partially activates Arch expressed in Sst-INs.

The several hour duration of the LTP obtained *in vivo* implies a protein synthesis-dependent mechanism;^39^ however, a definite conclusion would require testing the effect of protein synthesis inhibitors.

The finding that suppression of Sst-INs, but not PV-INs, enables LTP induction in BLA input from dmPFC, suggests that Sst-INs are distinct groups of GABAergic neurons, specializing in gating synaptic plasticity in remote inputs to BLA.

Like in the cortex, the Sst-INs axonal terminals in BLA target mostly the distal dendrites of PNs, whereas the PV-INs preferentially target somas and proximal dendrites.^40^^–^^43^ Then, a straightforward interpretation of our findings is that dendritic but not somatic disinhibition favors LTP in BLA. Nevertheless, the finding by Luthi’s lab that auditory cue activates PV-INs to suppress Sst-INs, and then aversive footshock suppresses PV-Ins, and Sst-INs altogether^22^ suggest that both dendritic and somatic disinhibition are involved in fear learning. Meanwhile, our findings indicate that dendritic disinhibition alone is sufficient for the facilitation of BLA inputs. However, because Sst-INs also inhibit other INs, including PV-INs, some of which form reciprocal connections,^43^^–^^45^ more complex mechanisms than simple dendritic disinhibition may contribute to the LTP after the suppression of Sst-INs.

A similar role of Sst-INs was reported in the somatosensory cortex, where Sst-INs gate LTP in the lemniscal sensory pathway and their suppression by PV- and VIP-INs “opens that gate” and allows LTP induction.^46^ The LTP gating by Sst-INs, however, is not a universal phenomenon throughout the brain. For example, in the hippocampus, the Sst-INs located in the oriens/alveus region of the area CA1 rather enhance LTP in the Schaffer collateral pathway by inhibiting GABAergic neurons in the stratum radiatum, thereby disinhibiting the CA1 principal cells.^47^ The PV-INs, in turn, appear to gate the hippocampal LTP, based on the finding of a stronger LTP in the model mice for the presymptomatic amyotrophic lateral sclerosis (ALS) and Alzheimer, in which a mutated NRG1 receptor Erb4 causes deficiencies of the PV-INs.^48^^–^^50^ Thus, the PV-INs and Sst-INs oppose each other in both BLA and hippocampus, yet the roles of each IN population in LTP are reversed between the structures. Given such region-dependency of the PV- and Sst-IN functional relationship, the disinhibition-assisted LTP in different target regions may require suppressing of different subclasses of GABAergic neurons.

Another technical aspect is the choice between chemogenetic and optogenetic suppression. While DREADD is highly effective in suppressing GABA release from INs even when they fire APs, the drawbacks for *in vivo* experiments are the long washout times and the off target-effects of chemogenetic ligands.^51^ In addition, in hM4Di transgenic mice, CNO would suppress all Sst-INs, including those outside the target region, and cause nonspecific effects, which can be avoided by employing viral vectors for region-specific expression of hM4Di.

The optogenetic suppression of INs circumvents these problems, but using two different wavelengths of light *in vivo* is more expensive and technically demanding. An additional drawback of the two-light design is that stronger yellow light interferes with LTP induction, apparently by desensitizing Chronos, as seen with the ReaChR,^52^ even at the levels that do not activate Chronos to release neurotransmitter. This nonspecific effect of the yellow light on a blue-light activated opsin is an artifact that needs to be considered along with other optogenetics artifacts carefully summarized in the recent reviews.^53^^,^^54^ Fortunately, the problem can be avoided by using blue light alone, which is sufficient for LTP induction, both *ex vivo* and *in vivo*, with Arch expressed in Sst-INs. This is because the blue light (470 nm) still activates Arch, even though at the 35% efficiency when compared to the optimal wavelength (560 nm).^55^ However, using the same light source for inhibition of INs and excitation of glutamatergic axons limits the freedom to deliver different stimuli to two neuronal populations. Perhaps, the single-color disinhibition assisted LTP can be improved further by replacing Arch with a blue-shifted inhibitory opsin, like a proton pump Mac, activated by the blue 470-nm light at about 60% of the maximum efficiency.^55^
