# Supplementary material for: Disinhibition-assisted long-term potentiation in the prefrontal-amygdala pathway via suppression of somatostatin-expressing interneurons
Source: Neurophotonics. 2020 Feb 14;7(1):015007. doi: 10.1117/1.NPh.7.1.015007 (PMC7019182; doi:10.1117/1.NPh.7.1.015007)
Supplement: Supplementary file 1 [file NPh_007_015007_SD001.pdf]

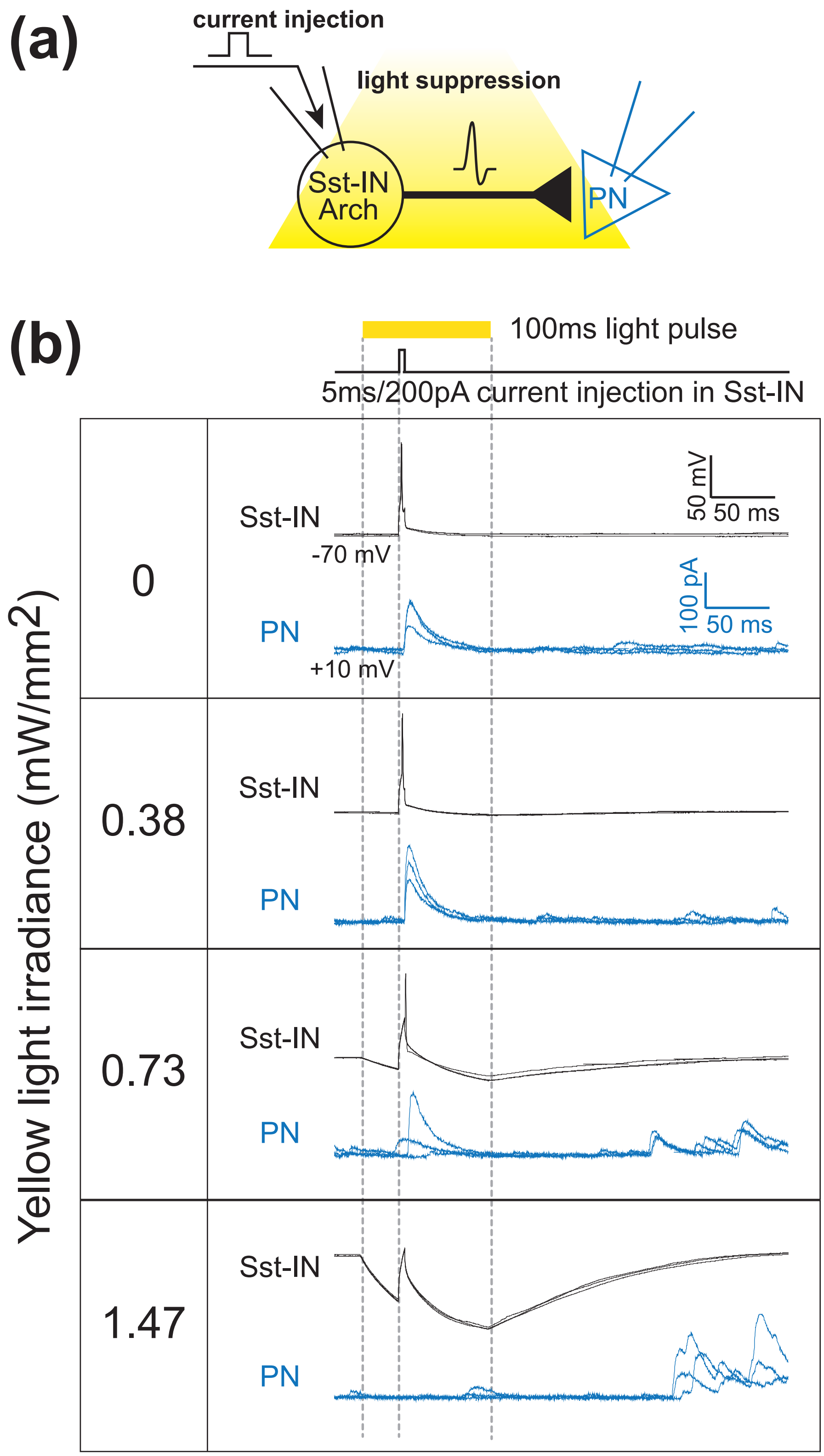

Fig. S1 Arch inhibits GABAergic output from an Sst-IN. (A) Experimental scheme. The 5 ms 200 pA depolarizing current was injected in an Sst-IN (at -70 mV) expressing Arch during the 100 ms pulse of yellow light, which started 30 ms before the current injection. Inhibitory postsynaptic currents were recorded from a connected principal neuron held at +10 mV. (B) Membrane potentials in the Sst-INs (black) and postsynaptic currents in the principal neuron (PN, blue) at different levels of yellow light irradiance.
