# Supplementary material for: Disinhibition-assisted long-term potentiation in the prefrontal-amygdala pathway via suppression of somatostatin-expressing interneurons
Source: Neurophotonics. 2020 Feb 14;7(1):015007. doi: 10.1117/1.NPh.7.1.015007 (PMC7019182; doi:10.1117/1.NPh.7.1.015007)
Supplement: Supplementary file 2 [file NPh_007_015007_SD002.pdf]

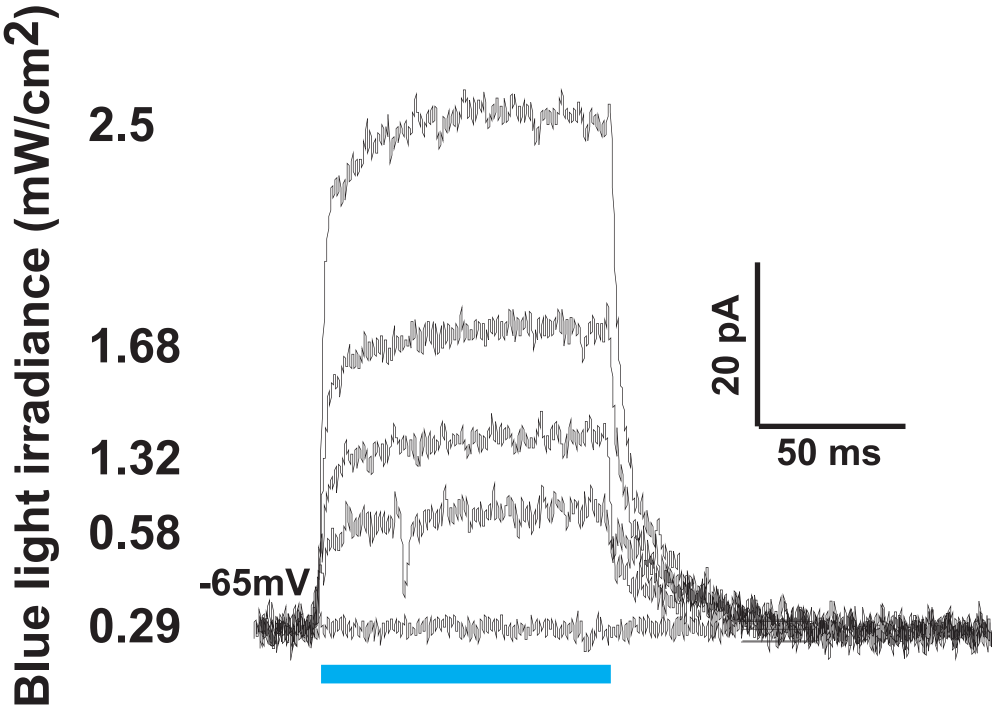

Fig. S2 Blue light elicits hyperpolarizing current in an Sst-IN expressing Arch. An example of the whole-cell recording from a BLA Sst-INs with Arch. The responses to 100 ms pulses of blues light of the indicated irradiance are shown.
