# Supplementary material for: Disinhibition-assisted long-term potentiation in the prefrontal-amygdala pathway via suppression of somatostatin-expressing interneurons
Source: Neurophotonics. 2020 Feb 14;7(1):015007. doi: 10.1117/1.NPh.7.1.015007 (PMC7019182; doi:10.1117/1.NPh.7.1.015007)
Supplement: Supplementary file 3 [file NPh_007_015007_SD003.pdf]

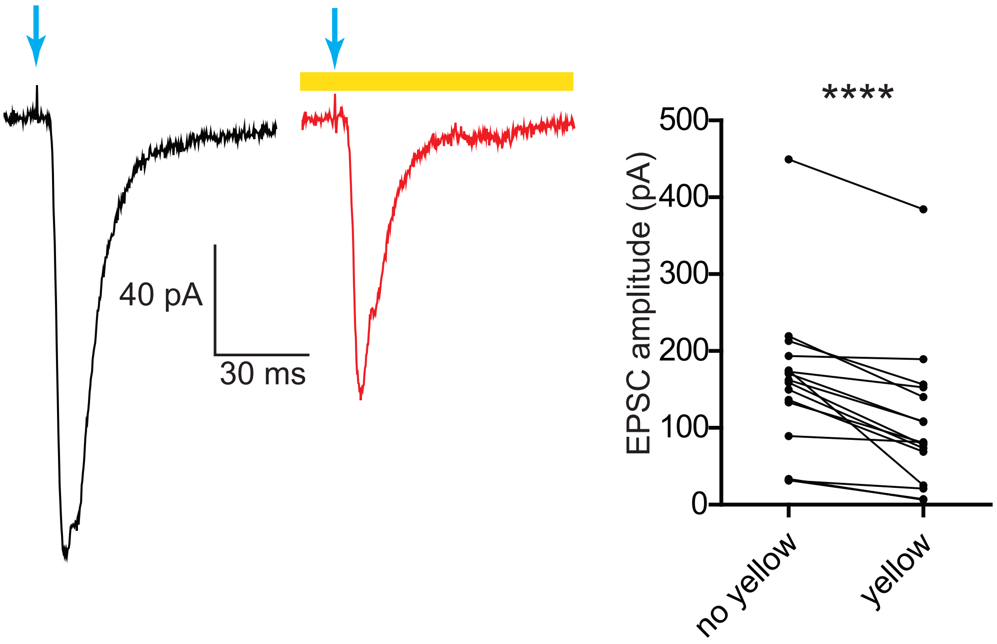

Fig. S3 Yellow light inhibits synaptic transmission induced via Chronos. Left: An example of EPSCs evoked in a BLA neuron by blue-light stimulation (blue arrows) of dmPFC axons expressing Chronos in the absence (black) and presence (red) of continuous yellow light at the irradiance of 0.24 mW/cm2. The traces shown represent averages of 5 sweeps. The sweeps in the absence and presence of yellow light were recorded in alternating order. Right: Summary of recordings from 16 BLA neurons in 5 slices from 2 mice. ****$p<0.0001$, paired t-test.
